# A Robotic Cognitive Architecture for Slope and Dam Inspections

**DOI:** 10.3390/s20164579

**Published:** 2020-08-15

**Authors:** Milena F. Pinto, Leonardo M. Honorio, Aurélio Melo, Andre L. M. Marcato

**Affiliations:** 1 Electronics Department, Federal Center for Technological Education of Rio de Janeiro, Rio de Janeiro CEP 20271, Brazil; milena.pinto@cefet-rj.br; 2 Electrical Engineering Department, Federal University of Juiz de Fora, Juiz de Fora CEP 36036, Brazil; aurelio.melo@engenharia.ufjf.br (A.M.); andre.marcato@ufjf.edu.br (A.L.M.M.)

**Keywords:** dam inspection, intelligent sensing, rule-based expert system, decentralized architecture, decision-making, 3D reconstruction, unmanned aerial vehicle

## Abstract

Big construction enterprises, such as electrical power generation dams and mining slopes, demand continuous visual inspections. The sizes of these structures and the necessary level of detail in each mission requires a conflicting set of multi-objective goals, such as performance, quality, and safety. It is challenging for human operators, or simple autonomous path-following drones, to process all this information, and thus, it is common that a mission must be repeated several times until it succeeds. This paper deals with this problem by developing a new cognitive architecture based on a collaborative environment between the unmanned aerial vehicles (UAVs) and other agents focusing on optimizing the data gathering, information processing, and decision-making. The proposed architecture breaks the problem into independent units ranging from sensors and actuators up to high-level intelligence processes. It organizes the structures into data and information; each agent may request an individual behavior from the system. To deal with conflicting behaviors, a supervisory agent analyzes all requests and defines the final planning. This architecture enables real-time decision-making with intelligent social behavior among the agents. Thus, it is possible to process and make decisions about the best way to accomplish the mission. To present the methodology, slope inspection scenarios are shown.

## 1. Introduction

In every large construction, there are contingency risks. Therefore, security constraints must achieve the highest standards. This is a sensitive situation, especially when considering mining and electric power generation, wherein slopes and dams are subject to enormous forces with real disruption danger. Among all types of inspection categories, the 3-dimensional surface deformation analysis (SDA) [1] is one of major importance. The SDA demands a very dense georeferenced point-cloud; each point must be compared with its correlative from previous inspections to find any possible displacement. The technical requirements for this type of visual inspection create a perfect environment for unmanned aerial vehicle (UAVs). However, considering the size and complexity of those constructions, it is prohibitive for one operator to deal with safety conditions, optimal inspection path, and the right distance, and to analyze the acquired data [2,3]. Therefore, the UAVs must be capable of cognitive-based decisions in order to optimize the quality and inspection time. This demand generates platforms with a high degree of autonomy carrying out tasks simultaneously, with less or none human intervention [4].

Inside each agent present on a mission, one of the most important characteristics is the decision-making process to generate this autonomy. Decision-making is one of the defining features of intelligent systems [5], and it drives the ability to accomplish the UAV mission goals. To make decisions, the UAV must gather the data from the sensors, and derive information and context. For example, images will be processed by computer vision software, such as deep learning, to recognize objects. The information about the objects and their positions, present at a given location, produces the context for a scene. Then, the UAV has to apply decision-making mechanisms using this information along with its knowledge from the past and goals to make decisions. As can be noticed, this is an organized process that uses many different structures.

Each UAV has its memory, process, and decision-making. However, some missions require different UAVs to work together to complete tasks. The coordination of multiple agents to accomplish a given goal also requires the integration of complex algorithms and methods working onboard and off the aircraft system, which must be engineered to develop specialized solutions to answer specific applications [6,7]. The decision-making process of each UAV has to be consistent and predictable to allow such integration. The literature shows several expert systems being applied into decision-making to find optimal solutions for specific multi-task problems. However, a few of those are integrated to solve general issues underlying synchronous cooperative operation. One way around this limitation is to couple an expert system to a cognitive approach that breaks down the problem into units, while making independent assumptions about their specialized knowledge. These units can range from low-level data interfaces, such as sensors and actuators, to high-level vision-based identification and decision-making machines. Another challenge with real-life cognitive-based systems is that the topology must represent, build, and debug each feature individually. Hence, the topology should have a scalability whereat each agent must be developed, tested, and integrated separately.

Thus, based on the problems mentioned above, this research highlights the importance of addressing the development of practical methods for inserting artificial cognition in UAVs and mission goals. Such a methodology would optimize the use of autonomous devices in critical decision-making processes, enabling that visual inspections be more precise, cost less, fast, and reliable.

### 1.1. Contribution

The main contribution of this work is the design and implementation of a cognitive-based architecture (CBA) designed to aid in large scale surface inspections. The CBA creates a high-level decision-making process through decentralized multi-objective reasoning. Therefore, each intelligent agent acquires raw data, processes it into information, and creates a solicitation for a given individual behavior. A supervisory agent is responsible for analyzing all solicitation and for defining the behavior of the system. For instance, it may analyze the current 3D point cloud, and in order to enhance quality, change the originally planned path.

Other contributions of this work are divided among application, architecture, and implementation as follows.

- An optimized approach to processing accurate visual-based decisions and3-dimensional surface reconstruction for slopes and dams.
- An organized and scalable paradigm to support decision-making and high-level cognition.
- An approach to enable the operator to be a human in-the-loop, with the sole obligation of analyzing the current inspection.
- A computational implementation of a decentralized architecture enabling autonomous UAV operation.
- A mechanism to provide qualified information in order to enable a human-in-the-loop operator to make confident decisions about the mission requirements.
- A real application of a cognitive-based architecture.
- Improvements in slope and dam inspections.

It is important to note that this research does not the focus in detail on each methodology, nor their effectiveness, but instead, it demonstrates the architecture and how they work together.

### 1.2. Organization

The remainder of the paper is organized as follows. Section 2 presents a brief review of the related work highlighting the state-of-the-art in cognitive architectures and aerial frameworks, and the use of UAVs in slope and dam inspections. Section 3 details the architecture and its foundations. Section 4 shows the proposed experiments with a proper discussion of the results. The concluding remarks and future work are conducted in Section 5.

## 2. Background and Related Work

Visual Inspections through UAVs require a combination of several specific and interrelated functionalities. In this context, a cognitive architecture is capable of not only creating intelligence-based behavior but also organizing all individual requirements in building blocks.

This section demonstrates the paper’s motivation by showing works using UAVs for dam inspections, the UAVs’ aerial architectures, and the most interesting cognitive systems topologies that could be used for the inspection problem. Finally, the related work shows that our improvements, i.e., to include new advances and to make aerial cognitive architectures suitable for civil inspections, are aligned with the state-of-the-art literature.

### 2.1. Slope and Dam Inspections with UAVs

Visual dam inspection is a crucial safety task [8] that, if not properly executed, may have a tremendous impact on infrastructure and human lives [9,10]. One crucial part of all inspection categories is the correct instrumentation and monitoring system, in which the main content is the 3-dimensional SDA. Typically, this activity uses 3D laser scanners [11]. However, this approach is time-consuming and difficult to apply due to the size and complexity of some structures. In recent years, the development in UAVs capabilities has dramatically changed this procedure [12,13].

More specifically, regarding dam inspection using UAVs, the literature shows several reports. For instance, Khaloo et al. [14] presented potential applications of UAVs along with photogrammetry to perform assessments and inspections of large structures, thereby generating point clouds with sub-millimeter accuracy. A similar approach was shown by Buffi et al. [15], wherein the images are also georeferenced by the application of markers in the inspected structure, increasing the inspection’s reliability even further.

The work of Özaslan et al. [16] exhibits an extreme application of UAV wherein the autonomous navigation system was applied to perform inspection inside a tunnel-like structure. This kind of scenario represents a fringe case due to environmental complexity. Despite showcasing the technology’s potential, autonomous navigation is very restricted to a specific task. They combined laser scanning and an inertial movement unit (IMU) to produce 3D localization and mapping features, allowing the UAV to determine its position in the structure.

The UAVs of the works mentioned above executed a predefined task without any tactical or strategical actions regarding the quality or optimization of the inspection, which is different from our implementation, wherein a cognitive-based topology capable of reactive, tactical, and strategic actions was developed.

### 2.2. Aerial Robotic Systems’ Frameworks

Different applications contain various requirements regarding the activities and levels of intelligence [17]. For instance, in search and rescue applications the high-level decisions are related to target detection, route evaluation [18], recognition [13,19,20,21], visual stability [22], team coordination [23], mapping [24], and autonomous landing [21]. However, more general decision-making is not the usual scope in this context. For instance, the presence of a decision-maker can enable the operation of UAVs in more dynamic environments, even without central planning or management. The aircraft can respond optimally to any environmental changes. The present research used a rule-based expert system method for decision-making with the capability of working autonomously in trained tasks.

There are many proposals creating approaches for the construction of intelligent systems. Aerostack [25] is one of the most prominent solutions. This framework presents a modular organization for command and control of autonomous heterogeneous teams. Moreover, this approach provides the incremental development and verification of a complex system using a mixed-reality concept. The operation was conducted onboard an aircraft. The tasks were path planning and collision avoidance. As a drawback, this framework is centered on sensorial data and it does not define a specific model for data storage. Besides, the architecture provides a base for improvements in human-in-the-loop (HITL), team coordination, and fully autonomous decision-making.

The intelligent vehicle control architecture (IVCA) [26] enables collaboration among multiple air vehicles. The IVCA is generic, but it aims at studying aircraft working cooperatively. This architecture is based on ontologies, and it does not contain any specification about hardware nor any experiment. The work proposed by Seleckỳ et al. [4] shows a fully UAV architecture for autonomous operation centered on sensor data. However, the learning capability was not implemented, and the hardware requirements depend on the framework extension.

An important characteristic when comparing different methodologies is how to use data for problem solving and decision-making. The work of Ball et al. [27] presents a review of the methodology employed by SOAR and ACT-R in the decision-making process; it uses expert knowledge and situational memory combined with rule-based logic to produce reasoning. Insaurralde et al. [28] showed an implementation wherein a centralized activity manager was responsible for optimal tasks’ distribution. The agent had an onboard decision-making algorithm in charge of path planning. Despite the satisfactory approach, agent planning was not prepared for decision-making in case of broader and generic missions. The proposed ARCog uses the expert’s knowledge compressed in rule-based expert systems and a protocol of symbolic messages to data and knowledge exchange among main blocks and layers. All required non-symbolic methods, such as computer vision, are enclosed to result in symbolic outputs. Thus, the reasoning performed within the architecture can be fully understood while analyzing the data flow.

Beyond data representation, it is also important to note that the incoming information about the world may be incomplete. Thus, the plans’ generation and decision-making process have to deal with this partial world representation around the agent. In general, symbolic reasoning mechanisms incorporate the incoming information with their background knowledge to determine an action based on the perceived reality. For instance, SOAR has been applied in this sense, as shown in [29].

Table 1 presents the advantages of the proposed architecture and the architectural differences among all related frameworks. It is possible to see that just one architecture other than the aerial robotics cognitive architecture (ARCog) has a mission different than path planning. Regarding the Aerostack (the complete architecture is found in the literature), it does not have the human-in-the-loop approach, and the mission is visual recognition. The human-in-the-loop is a very important feature for several reasons; the most important ones are related to the complexity of the mission and liability. When a computational system proposes a dam inspection and does not find any occurrence, it ensures structural safety. However, it is a very complex problem, with several nuances, making it irresponsible to transfer the responsibility from a human technician to a computational system. Therefore, the ARCog is a real and practical methodology designed to control all well-known conditions, to aid in structural inspection rather than takes the place of a human expert.

As one can notice, the inclusion of the human operator in the control loop is not a very common feature of cognitive architectures. Reference [31] presents a very comprehensive review of the automation enabled systems for visual infrastructure inspection. In the mentioned review, it becomes clear that despite the number of methods available and the many different automation levels, they do not include the human operator properly in the control loop.

When considering the human operator, the key feature is to model the human being into the decision-making system [31,32,33]. A simple approach missing from other architectures in Table 1 would be to consider the skill level of the operator, and the time that the mission takes to be executed. These two pieces of information can, for example, be used to set the level of automation the system can take. The skill level of the operator can be estimated, for example, by giving points for successful mission planning and execution and keeping score for each operator.

For example, the more advanced approaches considering the human operator in the control loop could use eye-tracking to determine the level of engagement with the task and mental models to estimate the operator’s level of stress. In this research, the operator’s skill level is taken into account when making decisions at the higher supervision level. Other works, such as [34], use similar strategies.

## 3. The Aerial Robotics Cognitive Architecture

### 3.1. General View

**Figure 1:**
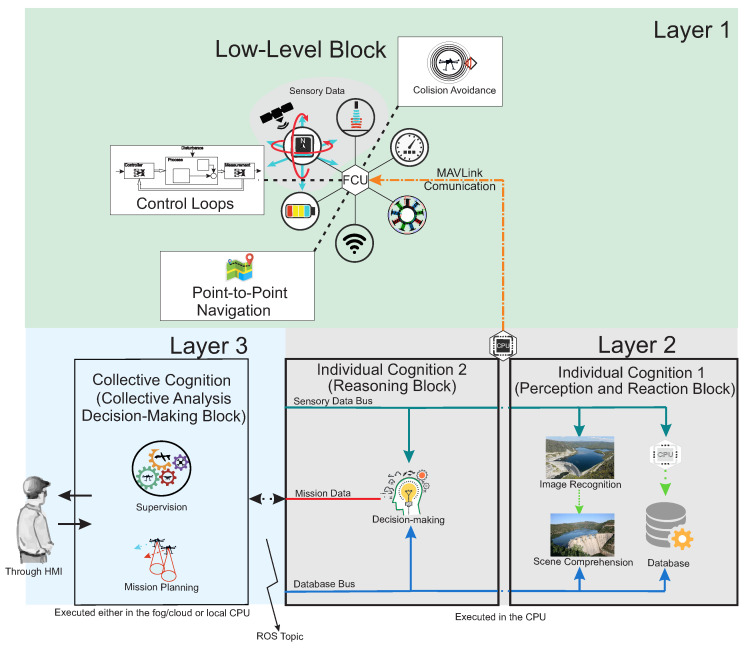
Main components of the ARCog.

This section presents an overview of the proposed multi-layer aerial robotics cognitive architecture (ARCog). The ARCog adopts a component organization where the blocks are inter-organized to match functional features, such as hardware interface, control loops [35], and path planning [36], among others. These characteristics ease the system’s engineering, besides improving performance and scalability. The functionalities and the internal tasks of the UAV can be seen as individual agents that need to be structured and supervised by a high-level management structure. Thus, the ARCog is based on the cognitive level paradigm [28]. Figure 1 exhibits the general overview of the architecture composed of four mains blocks organized in three layers.

The first layer consists of a basic perceptual system, such as an IMU and GPS processing, flight control loops [35], communication [37], payload evaluation [38], image acquisition, basic collision avoidance [39], and low-level navigation (point-to-point) [40] which are responsible for the *reactive* behavior. It has hard real-time requirements and it runs embedded at the aircraft’s flight control unit (FCU). Each inner agent processes its low-level data and passes the result information to the next layer.

The second layer is responsible for cognition capabilities. It has two functional blocks with soft-real-time requirements and moderate computational processing capabilities. They can be executed at a companion computer onboard at the aircraft or in a dedicated ground station (GS). More specifically, the first block of this layer (i.e., individual cognition 1) is responsible for a more advanced *tactical perception* by processing and analyzing the processed image recognition [30] and 3D reconstruction algorithms. The second block (i.e., individual cognition 2) is responsible for strategical reasoning, and it consumes the information passed by former layers and executes decision-making algorithms [41], e.g., rule-based expert systems [42]. The decision-making in this level has a database of scenarios in which the system takes advantage to make analogous actions for similar cases.

Unlike other architectures that focus on individual cognition, the aerial robotics cognitive architecture (ARCog) presents a third layer (i.e., the collective analysis decision-making block) responsible for the management of all individual knowledge of the agents (i.e., distinct internal processes) for collective analysis, and for *strategical* decision-making tasks. The supervision agent makes a decision considering all relevant information passed by other agents. If necessary, it can define new mission requirements, which are processed by a specific planner. Another relevant difference from other architectures is that the ARCog presents a human-in-the-loop topology. Considering that the system does not have all the necessary knowledge to inspect a given structure, it is possible to come across several unforeseen situations. These conditions must be analyzed by a human supervisor that can interact with the system by means of recommendation. Moreover, any unforeseen scenarios may be used to update the initial knowledge if the human operator deems it relevant. Therefore, the ARCog reduces but does not replace the human interaction [43].

The architecture runs multiple processes in a multitasking operating system through robotic operation system (ROS) communication methods. The mechanisms provided by the ROS are publish–subscribe schemes and request–reply services. The multitask operation is important to executing higher and lower processes with varied frequencies and computational times. For example, the image recognition algorithm demands soft real-time execution but with a greater computational cost.

### 3.2. Physical and Logical Implementation

**Figure 2:**
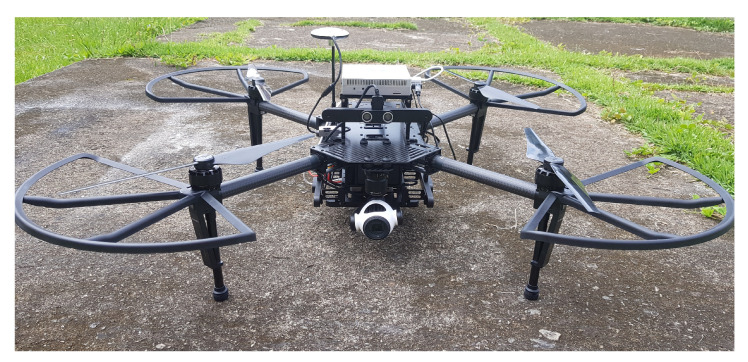
UAV Matrice used as part of the framework.

The ARCog was implemented using four different processing units. The first was an A3^®^ flight control unit (FCU) board that runs the basic low-level control. The second was a companion board computer equipped with a quad-core, Nvidia ARM CPU and 4 GB of ram. This equipment was chosen due to its low power consumption and fanless operation capability, and it was used to run the low-level tactical activities. The first two boards were embedded in the Matrice^®^ UAV, as shown in Figure 2. The third was a centralized laptop computer i7 7700 hq with 8 GB of RAM also used as a human–machine interface (HMI). The final processing unit was also a laptop computer with an 8 GB NVIDIA^®^ GeForce^®^ GRX1080 GPU. The architecture framework was built over the Ubuntu platform using ROS Kinect as a base for the development. The communication between the UAV and the ground station was built over Wi-Fi. The blue arrow indicates inboard communication, and the dashed gray illustrates ROS topic-based message passing.

**Figure 3:**
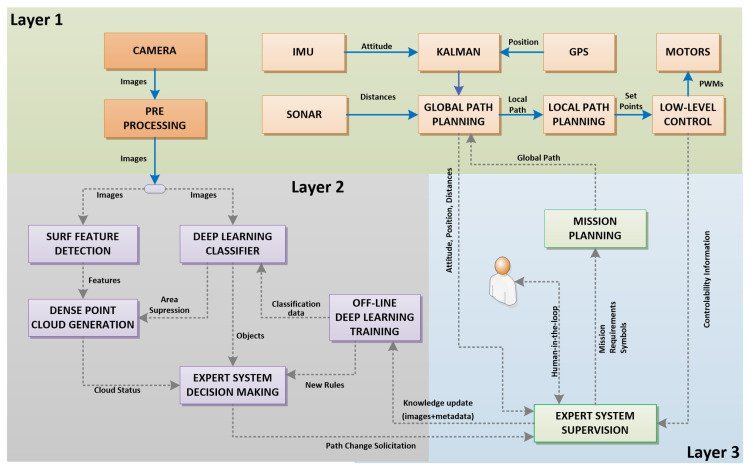
Block diagram showing the physical and logical implementation of the main components.

Figure 3 shows the details of the physical and logical implementation of the main components of the ARCog, initially shown in Figure 1. The background color indicates where each block runs; green is the low-level reactive block (layer 1) and is processed by the aircraft’s onboard FCU (light orange blocks) and companion board (dark orange blocks). The gray (layer 2) in the GPU laptop controls the collective strategical analysis and blue (layer 3) in the HMI laptop is responsible for the tactical cognitive decisions. Therefore, each level runs in a different unit. The gray dashed lines indicate ROS messages, and the solid blue ones are inner local communications.

#### 3.2.1. Low-Level Reactive Block

The low level-block is responsible for data gathering and reactive decisions. It uses the sonar, inertial movement unit (IMU) sensors, and GPS readings to process the global path planning (GPP). The GPP results in a local point-to-point (PtP) navigation considering the desired global path, current position, attitude, and detected obstacles. The local path planning reads the PtP, and accordingly with the current mission setup, defines the velocities and attitudes used as set-points for the low-level control. Finally, it is responsible for reading, treating saturation, and transmitting the camera images.

#### 3.2.2. Cognitive Tactical Level

The cognitive structure proposed in this work was designed based on Bloom’s Taxonomy of Learning Domains. This model was chosen to produce a hierarchical structure related to increasing levels of knowledge application. The framework structure provides an insight into the way human knowledge is built. For this reason, the taxonomy can also be used to organize an architecture’s components.

Individual cognition 1, shown in Figure 1 (i.e., perception and reaction block), contains methods that are more related to data processing, such as object recognition and scene comprehension. The image recognition block is a powerful tool to extract information from the environment. It implements two different methodologies. First, a pre-trained deep-learning algorithm [44] responsible for classifying a given image among three classes: conformity, non-conformity, and unknown. The conformity case is when the image corresponds to an expected aspect of a specific slope or dam. However, a non-conformity is when the system identifies a different color due to moisture, typical vegetation, crackles, and other well-known problems. An image is tagged as unknown if the classifier does not place it in the former two classes. This situation may represent, for instance, a non-critical scenario such as an animal/vegetation, or a critical one in case of an unseen structural problem. The second methodology is related to the 3D reconstruction algorithm. This work uses a real-time 3D monocular reconstruction library named COLMAP to generate the dense point cloud [45] and analyze the pixel density and shapes. An initial analyzed and verified 3D reconstruction is used as ground-true to find a 3D deformity. Then, the matching of common features between the current reconstruction and the ground-truth is performed. The system signals in case of a distortion larger than a selected threshold. An important observation is that this analysis is neglected in vegetation areas. Thus the deep learning classifier informs the dense point cloud generation block a given occurrence, and the region where the vegetation is identified does not account for reconstruction.

The individual cognition 2 layer (i.e., decision-making and off-line learning) takes the information from the previous block to produce a partial view of the world. The system builds its decisions based on predefined expert systems and previously collected scenarios. The usual methods for autonomous decisions are related to systems limited to extensive training over a big set of data. However, considering slopes and dams inspections, it is possible that each structure must have a different characteristic, and therefore, it is difficult or even impossible to start a cognition system with all necessary knowledge. Thus, the system must be operational with minimum intelligence and build its knowledge base over time. For that, it was used a rule-based expert system [42] that can be easily updated when required.

In a practical illustration, the individual cognition is responsible for analyzing the result of each algorithm from cognition level 1 (image classification and 3d reconstruction) and making high-level decisions. For instance, if common vegetation is found, it understands that this is a different situation, but does not represent any risk, so re-planning is not necessary. However, if a crackle is found, the system understands the danger, and it generates a solicitation to approximate the aircraft from the detected object.

Another situation regards the analysis of the 3D point cloud. If the system identifies a deformation from the original shape, it also requests a path-changing, but this time to increase the distance from the structure in order to get a more broad view. Finally, if the human operator identifies a new situation, he/she can require a knowledge update. This is an offline learning process, during which deep learning will associate new classes with the respective images. With a new image scenario, one or more rules can be added to the expert system.

The proposed expert system has seven heuristic rules, designed based on the cross expertise between how the image processing algorithms work and what is important in a visual inspection. Table 2 shows these rules that are ordered by priority. This means that if two situations are identified, just the top priority request is sent.

As can be noticed, in this level, there are two main structures. The first is responsible for detecting environmental characteristics and make this available as information. These structures are in the perception and reaction block. The second set of structures is responsible for taking immediate action onboard the UAV. Those parts are in the reasoning block. There are no functionalities overlapping in those blocks once the actual decision-making onboard of the UAV happens only in the second structure. The process in the perception and reaction block can be seen as the internalization of the data from the outside world into the UAV world. The decision-making can be applied once the information is internalized. This is similar to the process in other works, such as in [46,47].

#### 3.2.3. Strategic Collective Cognition Level

The third layer is responsible for strategic collective analysis and decision-making. The functionalities of this layer are encoded in a centralized system. It is responsible for reasoning all requests from other agents, and processes a unique collective behavior based on the drone’s current operational state. This analysis generates a new mission requirement, and if necessary, changes the current one by calculating a new global path.

Three agents send information to the supervisor agent to evaluate the methodology. The first one is the decision-making, shown in Section 3.2.2. The second one is the *global path planning* that gathers data from the Sonar and Kalman filter and sends the current aircraft state (position, distance map from obstacles, and remaining mission time). The third one is the low-level control that signals three different situations: low battery, rapid and robust switches in throttle indicate controllability issues, or if the throttles are close to the maximum performance, which may cause loss of sustainability. All of these conditions are critical, so have the highest priority. In the first case, due to the physical condition of the battery or a bad weather situation, the UAV reaches a critically low energy level. In the second case, a common possible cause is that, if the drone is too close to the slope, it may suffer from strong ascendant wind disturbances, which may be resolved by increasing the distance from the slope. The third case could be related to a lower, but not critical, battery state associated with wind conditions. Thus, if one of these two hypotheses happens, the secure action is to land the aircraft. Table 3 summarizes the possible requests from the low-level control agent.

Finally, the supervisor analyzes the requests, and along with the current aircraft state, decides if the desired action can be performed. In order to choose the best decision to make, a priority system has been defined wherein the low-level control takes precedence over the tactical level. The increasing and decreasing distance levels are parameters entered by the human operator at the start of the mission.

Considering the complexity of a civil inspection, a context-aware decision is fundamental. For instance, to define the critical state of a given crackle, it is necessary to know its evolution over time, position, shape, size, and mostly importantly, location on the structure. This is a very complex task, which differs from structure to structure, and without a proper dataset, it is challenging to deal with all situations autonomously, especially considering that a classification error may be critical. Therefore, an important aspect of the ARCog is the *human-in-the-loop* approach. The system coordinates the mission, assumes several tasks ranging from low-level control up to cognitive analysis, and passes to the human supervisor *information* rather than centralized data as a traditional system operational control does. With qualified information, the operator makes the best expert decisions about the mission requirements, improving not only the quality but the inspection time. For instance, if the human supervisor identifies a risky situation, he may request more detailed data from a given area or abandon the rest of the mission, regardless of distance, obstacle avoidance, controllability, mission time, and battery status. For the particular problem of slope and dam inspection, the system is also capable of ensuring its safety through low-level control, e.g., by performing the verification of the distance from the aircraft to the object in real-time, which would not be possible for a human operator.

Note that this level, as a hierarchically higher stage, has more broad power in reviewing the decisions made in the lower blocks. Here, the operator has the burden of evaluating conflicting interests according to the mission’s goals and making decisions. For example, if a crack is found, but there is not enough battery to perform a detailed inspection and finish the whole mission, what should be prioritized? This kind of question can be presented to the operator along with different path planning strategies while showing the time to complete each one. The operator is in charge of making the decision.

#### 3.2.4. Cognitive Mechanisms Underlying Decision-Making and Deep Learning

Cognition is defined as the process of acquiring knowledge, understanding, and thought [48]. In a robotic architecture, this process is performed using data from sensors that have to be processed to allow the production of useful information and enable decision-making. One of the most important sources of information is vision. However, the extraction of useful information from vision is a complicated task. Computer vision has to overcome many different issues, such as occlusion, change in lighting, and object appearance. Various methods have been applied to solve this issue, such as bag of words [49] and deep learning [50].

In this research, a machine learning methodology based on the deep learning algorithm is responsible for classifying a determined object in the image during the inspection using 3D data. As previously explained, this process will extract information from the images that can be used in the decision-making process. The main set of decisions to be made in-flight are related to the UAV inspection path. For example, deep learning has to identify and classify the slope’s and dam’s features to decide whether its path is suitable. There are three main classes involved during the classification process: conformity, non-conformity, and unknown. If a slope or dam is identified, this object is classified as conformity. Otherwise, non-conformity is when the system identifies other colors, such as typical vegetation and crack colors. In the case of none of them being identified, the system classifies it as an unknown.

Given these characteristics of the environment, it is possible to make decisions about the current path. The flow of information from classification and identification goes directly to the decision-maker. Many methods can be successfully employed to perform those kinds of decisions, such as expert systems [42], case-based reasoning [51], or even deep learning. However, due to the critical nature of the application, an expert system was selected once its behavior could be fully tested and evaluated. Consequently, the expert system will receive the objects and positions from the deep learning and decides if a request for path change solicitation to the expert system supervisor on layer 3 is proper.

Note that in case of any update in the system’s knowledge, two different processes must be applied. First, this information has to be relayed to layer 2 for the offline deep learning training. Second, the expert system knowledge must be updated to respond to the new inputs from deep learning properly. Decisions made in this layer are very localized. They are in resonance with the system’s goal to detect environmental characteristics. However, they do not take into account other environmental features, such as objects and available energy. Thus, a second expert system at layer 3 will receive those path changes requests and take into account UAV attitude, position, and distances to decide if the path changes requests can be fulfilled. Here again, many methods are suitable, and the expert system was chosen due to the full knowledge of its internal structure and operation. Learning at this stage also requires intervention where new rules are created into the system based on previous mission results.

As can be seen in the proposed architecture, the generation of knowledge and learning, and consequently, the decision-making is distributed among the many different layers. Different levels of decisions are required to take into account several aspects required by the complex activities. The rule-based decision-making methods selected can also be replaced in other applications if their requirements allow it.

#### 3.2.5. Human-In-The-Loop

One proposal of this work is to add human cognitive ability to the supervisory process. It is worth noting that any architecture can be changed to have human-in-the-loop in its processes. For instance, any autonomous drone mission has, in a simple way, a system that feeds the current status to an operator, who can interrupt or change the mission at any time. In this case, the human is responsible for data analysis, security conditions, trajectory optimization, inspection outcome, and, finally, re-planning if something different from what was expected happens. The information overload towards the operator results in a poor inspection quality. Considering scalability, it is impossible for a single operator to fully coordinate a fleet of autonomous vehicles to carry out an inspection mission.

The architectures presented in literature solve that problem by adding reasoning methodologies that took the operator’s place. However, they are general architectures and do not consider situations where the operator’s expertise is extremely complex to be replaced by a computer system but is fundamental for the precise mission’s accomplishment. In this case, the participation of a human at the supervisory layer is mandatory. The decision to add expert supervision to a given architecture depends exclusively on the problem’s complexity and the system’s reasoning capability. If the reasoning system is sufficient to capture all decisions made by a human operator safely, there is no need for HITL. However, complex situations such as structure surveys wherein rational decisions ensure the high quality of mission accomplishment, it is essentially an expert in the loop.

To support this activity, it is still necessary to apply an autonomous architecture to process acquired data through different cognitive reasoning levels to present only qualified information. The way of mapping and showing this information to an expert is also part of the proposed architecture.

Using the proposed architecture, the expert decision-making process focuses only on the inspection abstracting about the mission operationalization and execution. With this paradigm, the supervisor does not have to decide about obstacles, path optimization, energy saving, adverse flight conditions, aircraft integrity, or other mission-related issues. The only concern is to optimize the inspection quality based on high-level information.

Thus, the human in-the-loop is included in the interface methods to adapt and assist the operator continuously. Different missions may also need different types of assistance based on the problem’s complexity and the expert’s experience.

The expert system performs the supervision interfaces with the user to provide these functionalities on layer 3. This system has the function to assess the decision-making information on layer 2, images, and other metadata. It also has to determine the operator’s skill level, and if possible, his mental state, i.e., level of alertness of the user.

As can be noticed, there are many variables, and thus, closing the loop with the human operator can be quite challenging. Despite its difficulties, for complex activities, it can add an extra level of safety and efficiency. We devised a few examples to explain the role of the human in the loop at this level.

For the first example, for an alert operator, the system may only make decisions for critical actions and time-bound decisions. These are decisions that involve a large changes in path planning, for example, or to cancel a given mission due to battery availability. A second example is for an operator that is not so alert, in this case, the expert system supervision may alert the user about more sudden changes. These are changes such a light path change to increase picture quality during an inspection. In another example, when dealing with different skill levels, they may dose the amount of technical data available for the user when making a decision.

## 4. Results and Discussions

Two different inspection activities were performed to demonstrate the architecture capability with the following objectives:

- Testing the architecture’s ability to make autonomous actions towards the mission goals;
- Following the security requirements imposed on the system during the tasks execution and re-planning the mission when is necessary;
- Inspecting interesting points and structures through visual and 3D reconstruction analysis;
- Analyzing gains in terms of quality and execution time;
- Measuring human interference during routine inspections.

### Inspection in a Rocky Slope

The first inspected area was a rocky slope. The mission started at a close range looking for crackles on the surface. However, it is normal for the 3D reconstruction algorithm to present distortions due to several facts such as low feature identification. Hence, there were not many features to perform a good reconstruction causing deformities at the 3D point cloud. A common cause of this problem is that monotonic structures may not present good features in a close look. Therefore, this situation led to a *3D deformity* (rule 4 from Table 2) where the decision-making block requests the supervision to increase the distance. After analyzing the requests from all agents, the system setups a new path of 10 m away from the original one. The Mission Planning block evaluates this new path considering the camera field-of-view and remaining battery. After changing the original path, again, a *3D deformity* signal was accused, leading to a new path now 15 m away from the original. This time the tactical level had to emit a *continuous* signal, and the mission was fully executed. It is worth noting that, by choosing a more distant path from the slope, the search for crackle and moisture was neglected.

**Figure 4:**
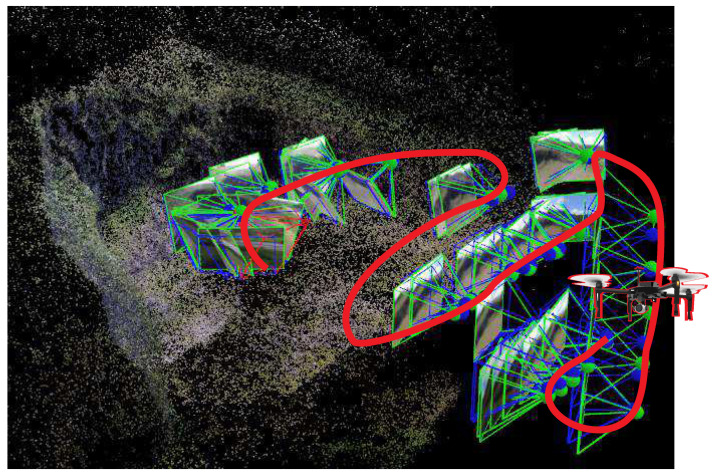
Positions of the cameras during the inspection and the UAV trajectory.

**Figure 5:**
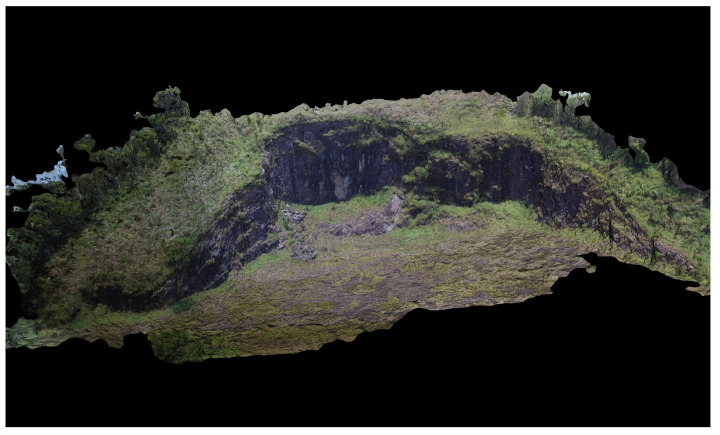
Reconstructed area.

Figure 4 shows the position of the camera during the mission and the trajectory followed by the aerial robot during its execution. This pattern is usually applied to perform inspection and produces high coverage of the full area. Figure 5 presents the reconstructed area as a final result of the inspection. Note that the rocks’ positions can be accurately observed.

The total mission time was 13 min, with no human intervention from take-off to landing. On the same weather condition, a human operator took 42 min, including one battery change, and the mission result was analyzed just after the mission.

The second task is the inspection of a hydroelectric dam. During this mission, the UAV is in charge of searching for vegetation around the dam. This is a typical operation where if the vegetation is growing too much a maintenance service is ordered. Thus, precise information saves time and costs.

**Figure 6:**
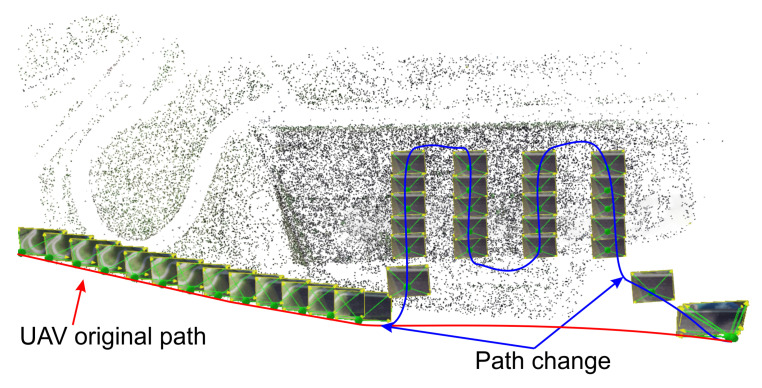
Path changing after recognizing points of interest in the inspection.

Initially, the mission planning algorithm creates a trajectory for the UAV, where the path is optimized to take enough images for reconstruction in minimum time. However, in the case that nonconformities (NCs) are detected, the decision-making system sends a request for path change to layer 3 (i.e., supervision). After processing, accepting, and re-planning the mission, the supervisory block sends the mission requirements to the global path planning to define a new mission closer to the dam. Images closer to the inspected area increase the level of details, but also increase the time for the mission. As soon as the NCs are out of the image, the path planning can resume the initial planning. Figure 6 shows the capability of the cognitive architecture of taking actions based on visual image recognition.

**Figure 7:**
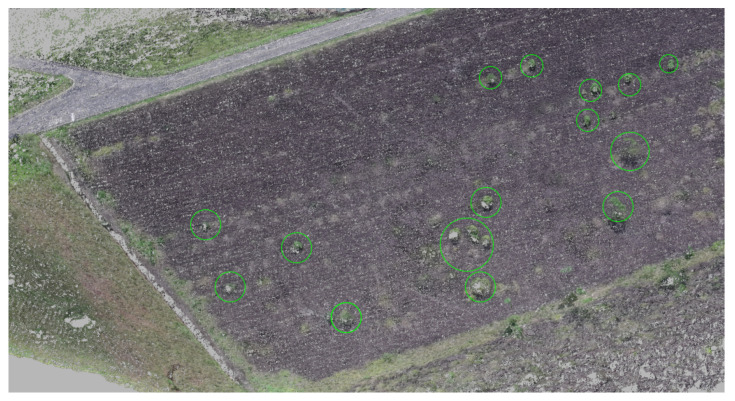
Recognized points of interest in the image.

**Figure 8:**
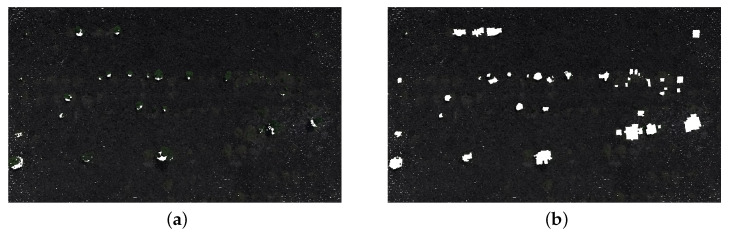
Results of vegetation suppression with (**a**) full vegetation and (**b**) using DL suppression.

In the end, it is possible to note the moment that no more NCs are recognized, the path returns to the original. Figure 7 shows the recognized NCs during the requested path planning change. As a result, it uses this information to suppress vegetation in the 3D point cloud, as shown in Figure 8a,b.

**Figure 9:**
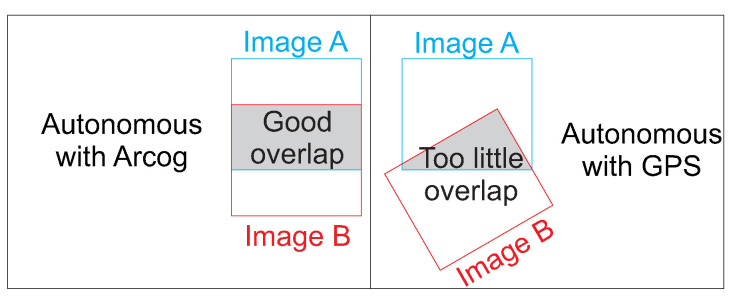
Characteristics of autonomous and manual inspections.

An interesting way of measuring the architecture’s capacity is comparing the results between the autonomous operation and the manual inspection. A great benefit of autonomous operation is the ability to capture data with a required amount of overlap and with adequate matching positions and orientations. Figure 9 shows a representation of these image acquisition errors. The autonomous image acquisition can also rely on non-specialized inspectors to produce consistent and accurate results.

Table 4 presents the quantitative results. Note that the inspection with ARCog demonstrates much more precision with higher resolution and higher mission time.

**Figure 10:**
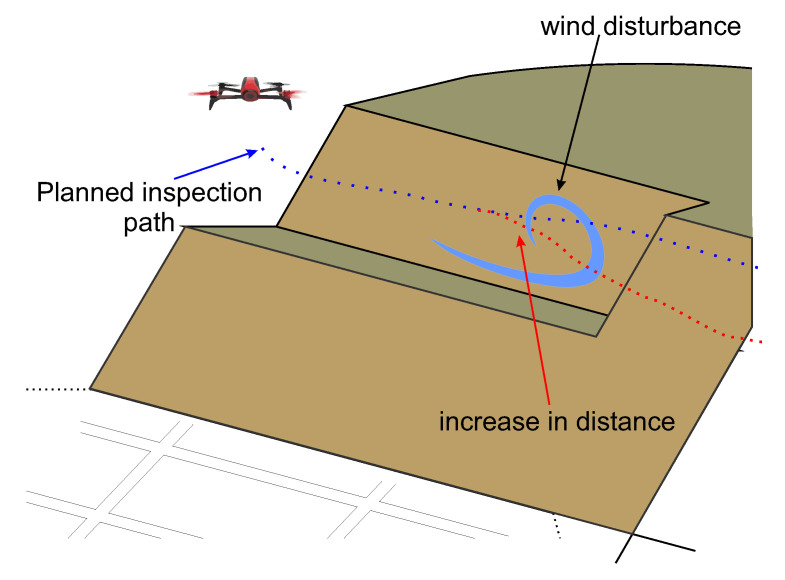
An aircraft suffering a wind disturbance during the inspection.

**Figure 11:**
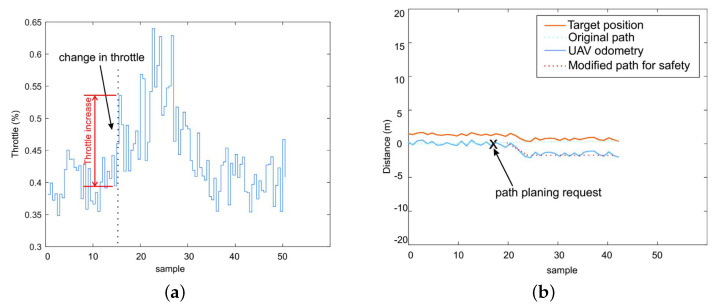
Modified UAV path based on safety: (**a**) UAV’s motors increasing their power to maintain the position. (**b**) Path changing during the wind disturbance.

Another important feature in the ARCog is the decision-making based on flight safety. This experiment shows the aircraft changing its path during the inspection. The UAV suffers from wind due to its approximation to the dam [52]. Figure 10 illustrates the wind disturbance suffered by the aircraft during the inspection. As a result of the disturbance, the motors increase the PWM switching amplitude to withstand the disturbance and keep the desired attitude. Figure 11b shows the UAV original path planning and the target position (i.e., dam) measured by the depth sensor in relation to the aircraft body frame. During a wind disturbance, the system requests for path planning, and the aircraft increases its distance from the dam. Figure 11a shows the change in throttle when this event occurred.

## 5. Conclusions and Future Work

Intelligent aerial robotic systems require a combination of several specialized and interrelated building blocks to reach autonomous operation capabilities. Many architectures for UAVs have been developed to guarantee a fully autonomous and safe operation. However, the autonomy level is often limited by a specific application or knowledge about the mission. Therefore, this research has described an organized, scalable, and decentralized architecture to support decision-making, such as interpretation and goal-oriented reasoning from the cooperation between high-level autonomous cognition systems and expert knowledge.

The experiments confirmed the successful use of the architecture in semi-autonomous missions and large scale visual and 3D surface inspections. The UAV presented autonomy in the decision-making process when recognizing non-conformities during the inspection and re-planning its mission to end the operation safely and with a high degree of information. A great advantage of the architecture is its low time consumption and flexibility in accessing difficult areas due to the use of UAVs. Besides, it leaves the human supervisor functioning as an expert in civil inspections just handling corrections in high-level decisions, thereby leading to better and faster results. Therefore, the cognitive approach with a human-in-the-loop has presented the ability to mix high-level processing capability, controllability, and safety with the extremely valuable knowledge of civil engineering. Another advantage is that the human supervisor does not have to know how to operate a drone, just analyze the results, and provide high-level commands.

This work opens the possibility of several future developments, such as uncertainty-based decision support with a fuzzy or Bayesian inferences process, the use of online reinforcement learning to train new behaviors during the mission, the application of context-aware methodologies to increase reasoning in the decision-making process, and the implementation of this methodology for more than one UAV, with different characteristics and sensors.

## Figures and Tables

**Table 1:** Advantages of the ARCog compared with related aerial robotics architectures.

Architectures	Features	
	Practical Experiments	Human-in-The-Loop
ARCog	Yes, Outdoor inspection 3D Inspection	Yes
FFIDUAS (2015) [10]	Yes, Outdoor path planning	No
IVCA (2014) [12]	No	No
STRL (2016) [17]	Yes, Outdoor path planning	No
Proactive (2015) [30]	No	No
Aerostack (2017) [11]	Yes, Outdoor and indoor Search and Rescue	No

**Table 2:** Expert system rules considering visual recognition analysis.

RULE	IF	REQUEST
1	unknown	human-assistance
2	crackle	decrease-distance
3	moisture	decrease-distance
4	3Ddeformity	increase-distance
5	bad-reconstruction	decrease-distance
6	vegetation	continue
7	nothing	continue

**Table 3:** Expert system rules for low-level control indexed by priority.

RULE	IF	REQUEST
1	low battery	land
2	maximum performance	land
3	switches	increase-distance

**Table 4:** Quantitative comparison between autonomous and manual inspections.

Parameter	Autonomous with ARCog	Autonomous with GPS
Average Resolution	21.3 points/cm^2^	9 points/cm^2^
Mean Error X(σ)	0.07 m	0.51 m
Mean GPS Distance(σ)	0.229 m	0.821 m
Mission Time	11 min	8 mins
